# Supplementary material for: Identification of photoperiod-induced specific miRNAs in the adrenal glands of Sunite sheep (Ovis aries)
Source: Front Vet Sci. 2022 Jul 22;9:888207. doi: 10.3389/fvets.2022.888207 (PMC9354845; doi:10.3389/fvets.2022.888207)
Supplement: Supplementary file 1 [file Data_Sheet_1.ZIP › Supplementary file/miRDeep2 file.zip!/miRDeep2 file/Know_Structure/SPLP42A3/oar-mir-369.pdf]

miRBase precursor : oar-mir-369
Total read count : 5751
oar-miR-369-5p read count : 1624
oar-miR-369-3p read count : 4125
remaining reads : 2

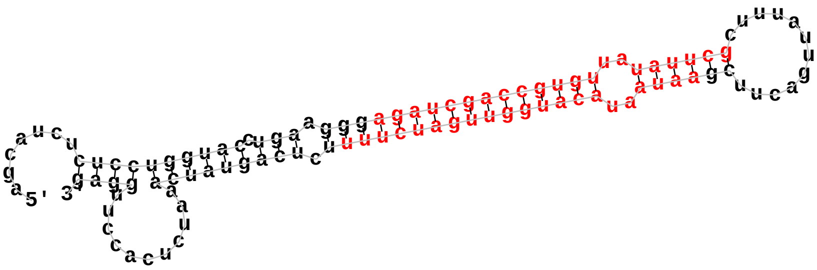

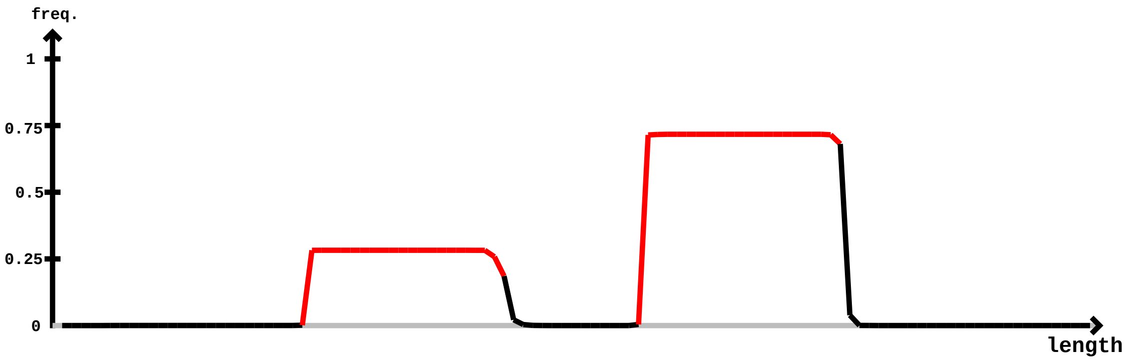

oar-miR-369-5p

oar-miR-369-3p

```
5'- agcaucucuccugguaccugaagggagaucgaccguguuauauucgcuuuauugacuucgaauaauacaugguugaucuuuucucaguaucaaaucucaccuuggag   -3'   exp
    .......((((((((((.(((.((((((((((((((((..((((((............))))))..)))))))))))))))).)))))))))...........))))   reads   mm   sample
    ....ucucuccugguaccugaaggg..................................................................................   1       0    seq
    .....cucuccugguaccugaaggg..................................................................................   1       0    seq
    ........................gagaucgaccguguuauauucg.............................................................   6       0    seq
    .........................agaucgaccguguuaua.................................................................   1       0    seq
    .........................agaucgaccguguuauauu...............................................................   139     0    seq
    .........................agaucgaccguguuauauuc..............................................................   417     0    seq
    .........................agaucgaccguguuauauucg.............................................................   935     0    seq
    .........................agaucgaccguguuauauucgc............................................................   103     0    seq
    .........................agaucgaccguguuauauucgcu...........................................................   18      0    seq
    .........................agaucgaccguguuauauucgcuu..........................................................   3       0    seq
    .........................agaucgaccguguuauauucgcuuu.........................................................   1       0    seq
    ..........................gaucgaccguguuauauucg.............................................................   1       0    seq
    ...........................................................gaauaauacaugguugaucuu...........................   1       0    seq
    ...........................................................gaauaauacaugguugaucuuu..........................   24      0    seq
    ............................................................aauaauacaugguugaucu............................   10      0    seq
    ............................................................aauaauacaugguugaucuu...........................   197     0    seq
    ............................................................aauaauacaugguugaucuuu..........................   3661    0    seq
    ............................................................aauaauacaugguugaucuuuu.........................   217     0    seq
    .............................................................auaauacaugguugaucuuu..........................   7       0    seq
    .............................................................auaauacaugguugaucuuuu.........................   3       0    seq
    .............................................................auaauacaugguugaucuuuuc........................   1       0    seq
    ..............................................................uaauacaugguugaucuuu..........................   2       0    seq
    ..............................................................uaauacaugguugaucuuuucu.......................   2       0    seq
```
